# A rare case of acute meningitis caused by *Moraxella osloensis*

**DOI:** 10.1111/cns.70011

**Published:** 2024-08-23

**Authors:** Yan Li, Guan‐Qing Wang, Xue‐Li Ma, Yan‐Bin Li

**Affiliations:** ^1^ The First Clinical Medical College Shandong University of Traditional Chinese Medicine Jinan China; ^2^ Department of Neurology, Shandong Provincial Qianfoshan Hospital The First Affiliated Hospital of Shandong First Medical University Jinan China; ^3^ Shandong Key Laboratory of Rheumatic Disease and Translational Medicine Shandong Institute of Neuroimmunology Jinan China; ^4^ The Second People's Hospital of Liaocheng Liaocheng China

**Keywords:** case report, meningitis, mNGS, *Moraxella osloensis*, treatment

## Abstract

Meningitis caused by *Moraxella osloensis* is rare and easily misdiagnosed clinically. Here, we report the first case of meningitis caused by *M. osloensis* in China by taking advantage of the metagenomic next‐generation sequencing technology in cerebrospinal fluid for pathogen screening. In addition, we extend the neurological signs, clinical symptoms, diagnostic methods, and treatment of this rare disease.

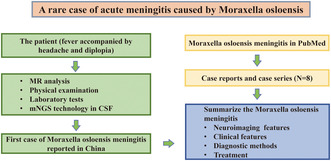

*Moraxella osloensis* is one of seven species in the genus *Moraxella*.^1^ It is an oxidase‐positive, catalase‐positive, and aerobic Gram‐negative coccobacillus known to be pathogenic to mollusks and uses nematodes as vectors.^2^ *M. osloensis* was common and the most frequent isolate from blood and cerebrospinal fluid (CSF) among clinical isolates of *Moraxella* spp.^3^ It has very rarely been reported to cause local or invasive infections. Here, we report a case of *M. osloensis* Meningitis in China, diagnosed by metagenomic next‐generation sequencing (mNGS). To the best of our information, this is the first report to reveal meningitis caused by *M. osloensis* in China.

## 1 CASE REPORT

The patient, an 18‐year‐old man, was hospitalized with a headache, fever, and diplopia. He was previously healthy, with no history of bird feces or soil contact. Seven days before admission, the patient experienced persistent acute headaches, primarily in the parietal, without any distinct cause. Moreover, the patient experienced a high fever of up to 40°C, mild nausea, and vomiting, but without diplopia, limb movement disorder, myalgia, chest tightness, or shortness of breath. Three days before admission, the patient sought local hospital help to manage the headaches and fever. Auxiliary serum blood cell counts examination revealed elevated white blood cell (WBC) count (Table 1); blood biochemistry was normal. No apparent abnormalities were observed on cranial CT and MRI. Antibiotic treatment of ceftriaxone and antiviral treatment of ganciclovir and ribavirin (about 3 days) were carried out in the local hospital, and the symptoms further deteriorated. Subsequently, the patient was transferred to our hospital. At this time, the patient developed a low‐grade fever accompanied by headache and diplopia.

**TABLE 1:** Some data of laboratory tests. Laboratory data for this patient.

Items	September 1, 2022	September 5, 2022	September 16, 2022
Peripheral blood			
WBC (10^9^/L)	12.48	10.66	n.a.
NEUT (10^9^/L)	9.46	6.39	n.a.
LY (10^9^/L)	n.a.	3.49	n.a.
CSF			
WBC (10^6^/L)	n.a.	115	15
Protein (mg/dL)	n.a.	27.7	19.9
Glucose (mmol/L)	n.a.	3.11	3.24
Chlorine (mmol/L)	n.a.	122	126.7
Opening pressure (mm H_2_O)	n.a.	380	230
Bacterial culture (5 days)	n.a.	(−)	(−)

Abbreviation: n.a., not available.

**FIGURE 1:**
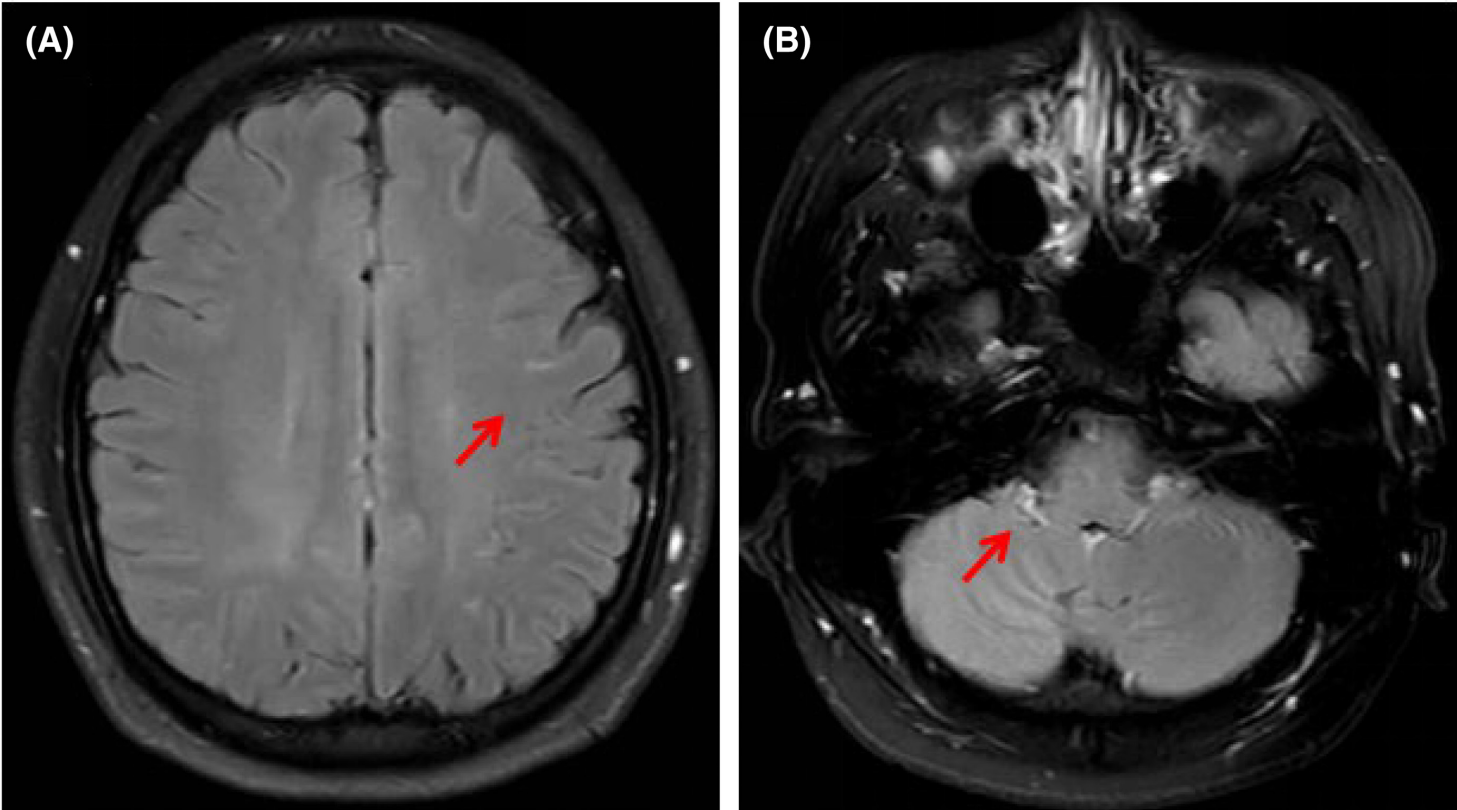
(A) Contrast‐enhanced T2‐weighted magnetic resonance imaging (T2WI) shows that leptomeningeal appears as enhancing curvilinear segments following the gyral convolutions of the bilateral cerebral hemispheres (arrow). (B) Contrast‐enhanced T2WI FLAIR showing the significant enhancement of the bilateral cerebellar leptomeningeal (arrow).

At admission, physical examination showed that the patient had clear consciousness, fluent speech, equal pupils, pupil light reflex is normal, and an inability to abduct the right eyeball; the muscle strength and tension of limbs are normal, and the bilateral Babinski sign was negative; signs of meningeal irritation, bilateral Kerning sign and Brudzinski sign were negative. An enhanced MRI of the brain region revealed abnormal enhanced meningeal pia of the bilateral cerebral and cerebellar hemispheres (Figure 1). The laboratory tests showed that there were WBC 10.66 × 10^9^/L (reference value range (r.v.), 3.5–9.5 × 10^9^/L), neutrophils (NEUT) 6.39 × 10^9^/L (r.v.: 1.8–6.3 × 10^9^/L), and lymphocytes (LY) 3.49 × 10^9^/L (r.v.: 1.1–3.2 × 10^9^/L) (Table 1). His blood biochemistry was normal except for the raised glutamic‐pyruvic transaminase level of 175.6 IU/L (r.v.: <50 IU/L) and glutamic oxalacetic transaminase of 42.4 IU/L (r.v.: <40 international IU/L) (we consider the side effects of ribavirin). His inflammatory biomarkers showed an elevated high‐sensitivity C‐reaction protein level of >10.7 mg/L (r.v.: <2.87 mg/L). His common virus detection in blood showed that toxoplasma IgM and IgG were lower than normal; rubella virus, cytomegalovirus, herpes simplex virus I + II, Epstein–Barr virus (EB virus), and parvovirus had lower than normal IgM but higher than normal IgG. T‐cell spot test tuberculosis infection (T‐SPOT.TB) and bacterial culture were negative in the blood. The first lumbar puncture in our hospital revealed the opening pressure was 380 mmH_2_O and the CSF transparent and clear, with a WBC 115 × 10^6^/L; Pandy's test was weakly positive; glucose (GLU), chloride ion, and protein levels were normal (Table 1); Determination of human cytomegalovirus and EB virus DNA was below normal value; Cryptococcus was not detected under a microscope; bacterial culture did not detect any bacteria; The acid‐fast staining was negative.

Considering the results of the symptoms of intracranial hypertension, CSF analysis, the high open pressure, and MRI findings the patient was initially diagnosed with meningitis. However, virus detection in blood and CSF (does not consider recent viral infections) and other pathogen detection did not prove which pathogenic microorganism was responsible for this patient's meningitis. We decided to place the CSF for further testing by mNGS. We treated this patient empirically with antiviral drug (acyclovir 500 mg, intravenous three times per day), decreasing intracranial pressure (mannitol), mecobalamin (500 mg, orally three times per day), and methylprednisolone (intravenous 80 mg/day); it did not significantly improve his condition.

Two days later, only *M. osloensis* (sequence number: 42) was identified in the CSF by mNGS; no parasites or other pathogens were found. The patient thus initiated broad‐spectrum antibiotic cefoperazone sodium/sulbactam sodium (Sulperazone®) (3 g, intravenous two times per day) therapy for 10 days. After 3 days on antibiotics, his clinical condition improved (no fever and a headache, better eye abduction than before). The CSF analysis showed a decreased WBC count that decreased to 15 × 10^6^/L (Table 1). The patient was discharged without complications on the 14th hospital day.

## 2 DISCUSSION

To the best of our information, this is the first report to reveal meningitis caused by *M. osloensis* in China. *M. osloensis* meningitis has been reported very rarely worldwide, with a total of eight cases in the literature through a PubMed search (Table 2).^4^, ^5^, ^6^, ^7^, ^8^, ^9^ These patients are mainly children and adults with underlying medical conditions, four of whom had susceptibility factors. Hereditary or acquired complement deficiency is frequently associated with meningitis caused by *Moraxella* spp.^7^ The patient, in this case, did not have an underlying disease, but unfortunately was not further examined for immunodeficiency.

**TABLE 2:** Summary of the clinical cases of *Moraxella osloensis* meningitis published in the last 50 years.

Reference	Gender	Age (years)	Underlying disease	Manifestation	Peripheral blood WBC (×10^9^/L)	CSF WBC (×10^6^/L)	CSF protein (mg/dL)	Diagnostic method	Treatment	Outcome
Hansen et al.^4^	F	1.5	Hydrocephaly and CSF shunt	Fever, meningeal signs, enlargement of the cranical perimeter	n.a.	1400	75	CSF culture	Methicillin; chloramphenicol; penicillin	Recovered
Berger and Kreissel^5^	M	4	Not found	Vomiting, somnolence, fever	14.3	14,850	325	CSF culture	Penicillin	Recovered
Fritsche et al.^6^	F	4	Not found	Fever, headache, cold, cough, skin sports	16.0	n.a.	16.4	CSF culture	Penicillin	Recovered
Fijen et al.^7^	M	15	Complement C8 deficiency	Fever, vomiting, petechiae, meningeal irritability, disturbance of consciousness	n.a.	82	n.a.	CSF culture	Penicillin	Recovered
Roh et al.^8^	F	4	Not found	Headache, fever, abdominal pain, vomiting	19.8	890	31	CSF culture; 16S rRNA gene sequence	Cefotaxime; ampicillin	Recovered
Roh et al.^8^	M	15	Not found	Headache, petechiae, neck stiffness	30.2	35,500	575	CSF culture; 16S rRNA gene sequence	Ceftazidime; netilmicin	Recovered
Roh et al.^8^	M	81	Pancreatic cancer and liver cirrhosis	Headache, petechiae, neck stiffness	6.9	9	75	CSF culture; 16S rRNA gene sequence	Cefotaxime	Recovered
Fox‐Lewis et al.^9^	F	31	Subclinical sinusitis	Rapid onset confusion, neck stiffness, headache	n.a.	92	325	CSF 16S rDNA PCR screening	Chloramphenicol; doxycycline	Recovered

Abbreviations: F, female; M, male; n.a., not available.

*M. osloensis* is difficult to identify because of the presence of several other species with similar phenotypic characteristics. Some cases were described with misidentification of *M. osloensis* as *Neisseria meningitidis*.^8^ Our patient presented with headache, fever, and cranial nerve injury, strongly suggested central nervous system infections. Viral meningitis was the first diagnosis; however, the use of antiviral therapy did not significantly improve the patient's signs and symptoms within 7 days of onset. Moreover, CSF and peripheral blood data failed to support this initial diagnosis. But the evidence for diagnosing bacterial meningitis is also insufficient, WBC counts are not very high and GLU, chloride ion, and protein levels were normal in the CSF (This is not the same as other cases) (Table 2), and no pathogens were found in CSF and blood culture (may be affected by early treatment).

Conventional pathogen diagnostic methods no longer meet diagnostic needs, we turned to mNGS for guidance. Because CSF is a sterile body fluid with a low bacterial load in the infected state, conventional microbiological CSF tests may be negative, mNGS is a new microbiological test that offers advantages over conventional tests in identifying the pathogens of encephalitis and meningitis.^10^ The mNGS shortened the time of diagnosis in the patient so that a guided accurate medication reduced the risk of disease progression. Therefore, mNGS should be applied as soon as possible, especially for patients for whom traditional diagnostic methods have not been effective and for infections for unknown reasons.

In most of the reported cases, *M. osloensis* was susceptible to penicillin and cephalosporins (Table 2). There have, however, been reports in the literature of strains of *M. osloensis* that have developed resistance to penicillin.^4^ If allergic to either of these drugs, chloramphenicol and doxycycline are also effective.^9^ All reported patients recovered from the infection after appropriate antibiotic treatment. Our patient was empirically treated with cefoperazone sodium/sulbactam sodium (Sulperazone®). Like the other reported cases, he recovered completely.

In summary, meningitis caused by *M. osloensis* is rare and is easily misdiagnosed clinically. This report highlights the benefits of mNGS technology in CSF for pathogen screening and expands the clinical symptoms and treatment for this rare disease. More research is needed to prove what susceptible groups are more likely to be infected with *M. osloensis*.

## AUTHOR CONTRIBUTIONS

Yan Li performed the data acquisition and wrote the manuscript. Xue‐Li Ma collected radiological images. Guan‐Qing Wang and Yan‐Bin Li reviewed the manuscript and provided critical revision. All authors contributed to the study and approved the submitted version.

## FUNDING INFORMATION

This study was supported by grants from the Nature Science Foundation of Shandong (ZR2023MH127) and the Academic Promotion Programme of Shandong First Medical University (2019QL013).

## CONFLICT OF INTEREST STATEMENT

The authors declare no conflict of interest.

## INFORMED CONSENT

We obtained patient permission and informed consent for publishing their information and images.

## Data Availability

The data that support the findings of this study are available from the corresponding author upon reasonable request.
